# Immigrant Arrival and Tuberculosis among Large Immigrant- and Refugee-Receiving Countries, 2005–2009

**DOI:** 10.1155/2017/8567893

**Published:** 2017-03-23

**Authors:** Zachary White, John Painter, Paul Douglas, Ibrahim Abubakar, Howard Njoo, Chris Archibald, Jessica Halverson, John Robson, Drew L. Posey

**Affiliations:** ^1^Division of Global Migration and Quarantine, National Center for Emerging and Zoonotic Infectious Diseases, Centers for Disease Control and Prevention, Atlanta, GA, USA; ^2^Department of Immigration and Border Protection, Sydney, NSW, Australia; ^3^Public Health England and University College London, London, UK; ^4^Centre for Communicable Diseases and Infection Control, Public Health Agency of Canada, Ottawa, ON, Canada; ^5^Immigration New Zealand, Wellington, New Zealand

## Abstract

*Objective*. Tuberculosis control in foreign-born populations is a major public health concern for Australia, Canada, New Zealand, United Kingdom, and the United States, large immigrant- and refugee-receiving countries that comprise the Immigration and Refugee Health Working Group (IRHWG). Identifying and comparing immigration and distribution of foreign-born tuberculosis cases are important for developing targeted and collaborative interventions.* Methods*. Data stratified by year and country of birth from 2005 to 2009 were received from these five countries. Immigration totals, tuberculosis case totals, and multidrug-resistant tuberculosis (MDR TB) case totals from source countries were analyzed and compared to reveal similarities and differences for each member of the group.* Results*. Between 2005 and 2009, there were a combined 31,785,002 arrivals, 77,905 tuberculosis cases, and 888 MDR TB cases notified at the federal level in the IRHWG countries. India, China, Vietnam, and the Philippines accounted for 41.4% of the total foreign-born tuberculosis cases and 42.7% of the foreign-born MDR tuberculosis cases to IRHWG.* Interpretation*. Collaborative efforts across a small number of countries have the potential to yield sizeable gains in tuberculosis control for these large immigrant- and refugee-receiving countries.

## 1. Introduction

Tuberculosis is one of the world's largest public health challenges. Approximately one-third of the world's population is infected with tuberculosis (TB), and an estimated 1.5 million people die from the disease each year [1]. The World Health Organization (WHO) established targets for TB control by 2035 [2]. These goals include targets for rates of diagnosis and treatment completion rates for persons inside a country [2]. However, migration is a factor that provides challenges for meeting targets as well as opportunities for control, particularly for countries with low incidence for TB [3].

Worldwide, there are approximately 232 million international migrants, or 3.2% of the world's population [4]. Although western, industrialized countries receive a large percentage of international migrants, other parts of the world also receive a large number of international migrants. Parts of the world in which >10% of the population comprises international migrants include the Gulf countries, Eastern Europe, and even a few countries in Africa. Pertaining to TB, many of the top source countries for international migrants are also countries with a high burden of TB. For example, the top source country for international migrants in 2013 was India, a country with the highest burden of TB [1]. Other top countries for both international migrants and tuberculosis cases include the Russian Federation, China, Bangladesh, Pakistan, the Philippines, Afghanistan, and Indonesia.

Several of the world's largest immigrant- and refugee-receiving countries (Australia, Canada, New Zealand, the United States, and the United Kingdom) participate in the Immigration and Refugee Health Working Group (IRHWG), which aims to develop collaborative approaches for immigration and refugee resettlements, including TB. A majority of the TB cases in these countries are individuals who were born abroad, and the threat of multidrug-resistant (MDR) and extensively drug-resistant (XDR) TB is of significant concern [5–9]. Each participating country currently conducts TB screening overseas for immigrants (persons applying for permanent residency) and refugees; all members, except the United States, also perform TB screening overseas for persons who will live in the receiving country > 6 months, also known as long-term visitors [10–14].

To address modern TB threats, each IRHWG country is in the process of improving TB prevention-control efforts, especially as it relates to immigrant and refugee populations with high burden of TB. To better identify the primary source countries that contribute the largest migrating populations and most TB cases to the group as a whole, we conducted an analysis of arrivals and TB case diagnoses.

## 2. Methods

We analyzed data on TB cases and arrivals to Australia, Canada, New Zealand, the United Kingdom, and the United States. The arrival data from each IRHWG country include long-term visitors, immigrant arrivals, and refugee arrivals; the arrival data were obtained through the immigration bureau for each country. The TB data are comprised of an in-country diagnosis of foreign-born TB cases; the TB case reporting came from the National Reporting Agencies in each IRHWG country. The authors acknowledge that the TB cases in this analysis are not directly linked to the immigration data presented.

All TB case data were stratified by year, birth country, and type of TB (treatment-sensitive or MDR). For analysis purposes, cumulative totals from 2005 to 2009 were utilized to determine the total volume of immigrants and diagnosed tuberculosis for each IRHWG country. Microsoft Excel and JMP 9 were used to construct tables in order to compare relationships among immigration totals, TB cases, and MDR TB cases. The top 20 source countries for total immigrants, TB cases, and MDR TB cases were determined using Microsoft Excel. Bar graphs were constructed using Microsoft Excel and R statistical software and tables made from Microsoft Excel and JMP.

Because a goal of this analysis is to develop a perspective as to which source countries are the largest ones for the IRHWG members as a whole, irrespective of the numbers of arrivals or cases, we calculated the average of the percentages for each source country. This was calculated for arrivals, foreign-born TB cases, and foreign-MDR TB cases. The calculations in the bar graphs display the burden (immigration volume, TB case volume, and MDR TB case volume) for each member country, as well as the burden to the group as a whole. For example, the average of each country's proportion of TB cases born in specific countries is shown in Figure 2 as a bar graph. Each country's proportion to the total TB burden is also represented in the graph.

*Ethical Considerations*. Ethical approval and informed consent were not required for this analysis.

## 3. Results

**Figure 1:**
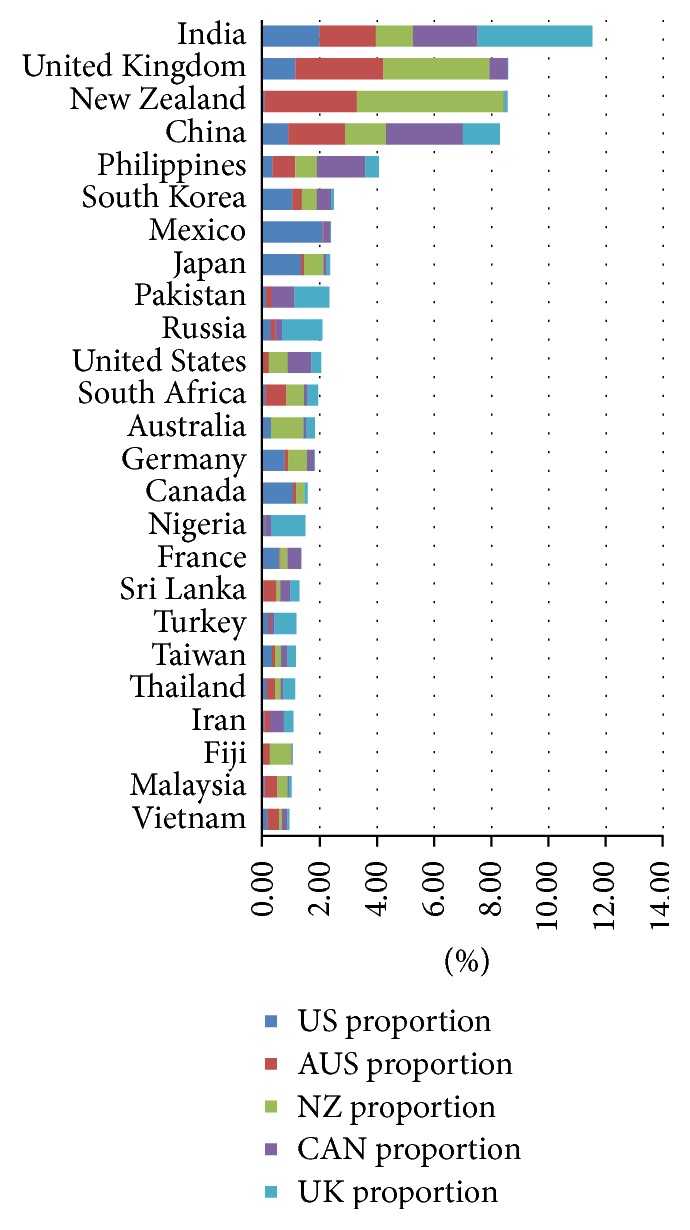
Combined averages of arrivals, 2005–2009.

**Figure 2:**
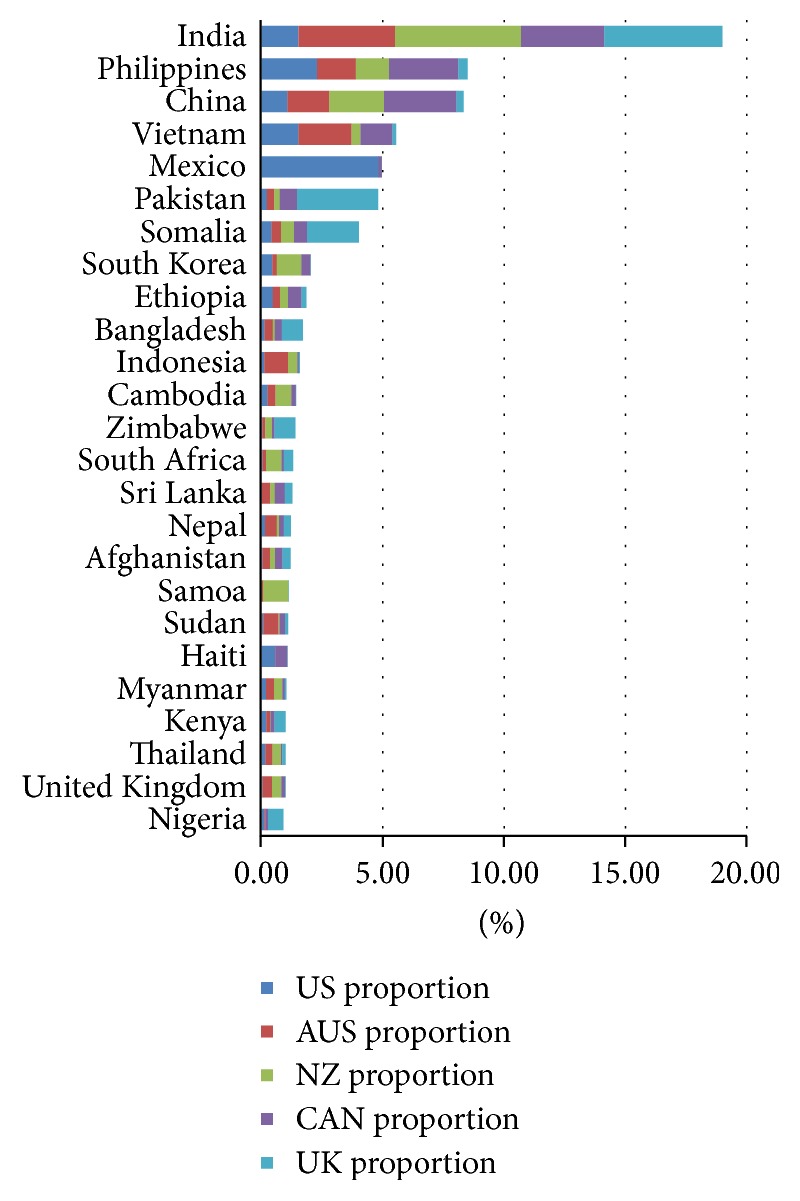
Combined averages of foreign-born TB cases, 2005–2009.

**Figure 3:**
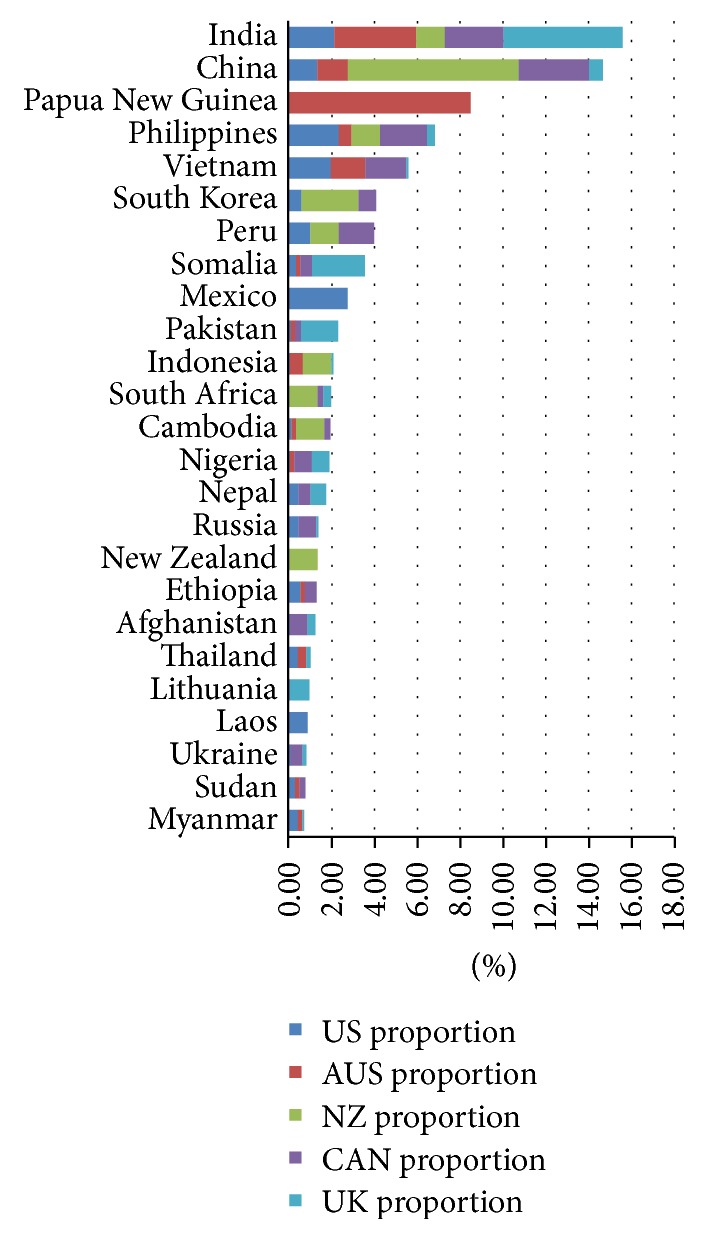
Combined averages of MDR TB cases, 2005–2009.

Between 2005 and 2009, there were 31,785,002 arrivals, 77,905 TB cases, and 888 MDR TB cases notified in these five countries. The data in Figures 1–3 represent the averages of each source country in respect to total arrivals, total TB cases, and total MDR TB cases from 2005 to 2009. The top five source countries for combined adjusted total arrivals (Figure 1) included India (11.5%), United Kingdom (8.6%), New Zealand (8.6%), China (8.3%), and the Philippines (4.1%), while the top five source countries for combined adjusted total tuberculosis cases (Figure 2) were India (19.0%), the Philippines (8.5%), China (8.3%), Vietnam (5.5%), and Mexico (5.0%). Similarly, the top five source countries for combined adjusted total MDR TB cases (Figure 3) were India (15.6%), China (14.7%), Papua New Guinea (8.5%), the Philippines (6.8%), and Vietnam (5.6%).

Data from the analysis reveal that India, China, Vietnam, and the Philippines supplied the majority of diagnosed TB counts. These four countries accounted for combined adjusted 41.4% of the total foreign-born TB cases (Figure 2) and 42.7% of the foreign-born MDR tuberculosis cases (Figure 3). Of these, India was the leading source country for arrivals (11.5% (Figure 1)), TB cases (19.0% (Figure 2)), and MDR TB cases from 2005 to 2009 (15.6% (Figure 3)).

Primary source countries for total immigrant arrivals, total TB cases, and total MDR TB cases varied for each of the five countries (Tables 1–3). Although multiple similarities were observed, the data reveal that four of the IRHWG countries have a specific source country/countries that contributes a significant amount of TB cases without affecting the group as a whole. Examples of specific source contributors include Papua New Guinea (3.4% of foreign-born TB cases and 42.4% of MDR TB cases) for Australia; Samoa (5.3% TB cases) for New Zealand; Somalia (10.6% TB cases and 12.3% MDR TB cases) and Pakistan (16.7% TB cases, 8.7% MDR TB cases) for the United Kingdom; and Mexico (24.1% TB cases and 13.7% MDR TB cases) for the United States (Tables 1, 2, and 3). Canada received the majority of cases from the four largest source countries (India, the Philippines, China, and Vietnam) but did not have a unique source country that was uncommon to the other members. In this analysis, instead of grouping countries by regions, we examined data for the individual source country to understand their specific impact on our immigration and TB programs.

## 4. Discussion

In this analysis, India, China, Vietnam, and the Philippines contributed the largest proportion of foreign-born TB cases to Australia, Canada, New Zealand, United Kingdom, and the United States as a whole during 2005–2009. The data used in this analysis is a couple of years old, but the immigration and TB trends are similar to the results of this analysis. The four countries accounted for 41.4% of combined adjusted foreign-born TB cases and 42.7% of the combined adjusted foreign-born MDR TB cases during this period of time. Each of these four countries is also included in the 2015 WHO list of high-burden countries with respect to TB [1].

However, these data also highlight the unique source countries for each of these countries, which illustrate differences in migration patterns. These unique situations may reflect factors related to geography, such as Mexico as a source country for the United States, or issues related to longstanding historical ties, such as Pakistan for the United Kingdom.

Although each country publishes immigration and TB surveillance figures, and the importance of foreign-born TB cases in industrialized countries has been described [15], this is the first comparison of immigration and TB data among large immigrant- and refugee-receiving countries to our knowledge.

The results of this analysis also mirror global comparisons over time. Just as the global trends in international migration have increased over time, so the volumes of arrivals have increased too during the study period. The growing importance of Asian arrivals, in particular, is also reflected in this analysis. In the United States, for example, the highest proportion of immigrants to the United States transitioned from Hispanics to Asians in 2009 [16].

The results also reflect global trends in TB, as many of the top birth countries for TB cases are also WHO high-burden countries. Although only five receiving countries are included in this analysis, the similarities (Figures 1–3) underscore the need to develop collaborative strategies to address the burden of TB in migrating populations [17].

During the period of this analysis, an average of 6 million persons were admitted annually to the five participating countries. Currently, approximately 2 million applicants for migration status to these five countries are screened for TB annually; the foreign-born TB cases which occur may or may not be discovered through the screening process. These examinations are performed by >2,000 panel physicians worldwide. Panel physicians are medical doctors who have agreements with each country to conduct the migrant medical exam overseas. Each of the IRHWG countries require overseas TB screening for all immigrants and refugees and each except for the United States routinely requires screening for long-term visitors. Moreover, all but the United Kingdom have historically required this screening [18]; following a successful pilot program in a limited number of countries, the United Kingdom began a targeted overseas screening program in 2012 for applicants from high-incidence countries [19]. Screening applicants for TB is very effective at preventing importation of active TB cases into the receiving countries [20, 21].

Managing a modern overseas TB screening program for migration and refugee resettlements results in development of laboratory and treatment capacity. The TB screening algorithms of IRHWG countries are similar in that they either require (United States) or use (Australia, Canada, New Zealand, and United Kingdom), where available, TB cultures for those suspected of having active pulmonary TB, drug susceptibility testing (DST) on positive isolates, and treatment delivered as directly observed therapy (DOT) prior to entry [10–14]. Since 2007, implementation of the US program's culture and DOT requirement has resulted in additional culture and DST laboratories as well as increased training for personnel involved with TB control [21, 22].

While each of these country's efforts in managing TB screening programs is invaluable for reducing importation of TB, they should also be leveraged to assist with control efforts within source countries. TB elimination in receiving countries is difficult without addressing TB in foreign-born populations [23]. For this reason, it is hoped that improved linkages between panel physician activities and TB control efforts within their countries would benefit the migrants and others in their source populations. This analysis helps demonstrate the fact that because panel physician volumes are large in key source countries, these countries are uniquely positioned to have their investments in the screening program also contribute to local control efforts [24]. And this analysis helps determine for which countries that contribution could be most needed. Additional benefits to screening programs could possibly be achieved by having panel physicians develop relationships with their TB controllers, share information on their experiences, share laboratory capacity, and comanage TB cases where DOT capacity is scarce.

The authors expected India to be a common source country but were surprised that the average number of cases was more than twice as high as that for the next two countries, the Philippines and China. The participating countries have collaborated closely with India, and this country was the first country for implementation of the United Kingdom screening program. However, the results of this analysis highlight the importance of collaborations with India for migrants, as well as for helping with overall TB control in India, since it is the highest-burden country for TB worldwide.

In many instances, TB treatment for those who travel to the US may not occur until years after they have arrived. While the data in this analysis shares source country of the migrants, it does not automatically imply importation. Individuals may acquire TB infection during travel or in the United States upon arrival.

Although this analysis yields important findings regarding migration and TB, there are some limitations. First, data for this analysis were only requested from 2005 to 2009, which is a short period of time. If additional years were observed for the analysis, additional trends for each country and the group as a whole might have been exposed. However, the data from these five countries for subsequent years suggest similar burdens of arrivals and diagnoses. Second, the analysis only included data for five countries. While these are some of the largest immigrant-receiving countries, additional future analyses should include countries outside the group. The analysis could have used data to separate the arrivals and TB cases by visa types for each receiving country. This would allow stratification of the arrivals and cases by populations to see which had the highest TB rates within each country. It is also important to note that the TB data were not stratified by time since arrival. Thus, the potential exists that trends among this subset may be slightly different. Finally, while this analysis can be used to help participating countries in the management of their screening programs by indicating which resources could be targeted, a cost-effectiveness review of screening programs on potential collaborations was beyond the scope of this analysis.

Australia, Canada, New Zealand, the United Kingdom, and the United States are the largest immigrant- and refugee-receiving countries in the world and are currently collaborating on preventing importation of TB into each of their countries. Joint efforts in a small number of high-burden countries can help prevent importation of TB cases and also contribute to control efforts within source countries.

## Figures and Tables

**Table 1:** Top source countries for arrivals, 2005–2009.

Australia			Canada			New Zealand			United Kingdom			United States		
Country	Number	%	Country	Number	%	Country^*∗*^	Number	%	Country	Number	%	Country	Number	%
New Zealand	114496	16.1	China	167658	13.4	United Kingdom	69316	18.6	India	2044222	20.1	Mexico	2021541	10.5
United Kingdom	109492	15.1	India	140599	11.3	China	26787	7.2	Russia	713690	7.0	India	1913293	9.9
China	70356	9.0	Philippines	105314	8.4	India	24180	6.5	China	656664	6.5	Japan	1294620	6.7
India	70173	9.9	United States	51594	4.1	Australia	21224	5.7	Pakistan	616792	6.1	United Kingdom	1096613	5.7
Philippines	28053	4.0	Pakistan	49713	4.0	Fiji	14361	3.9	Nigeria	605557	6.0	Canada	1027787	5.3
South Africa	26175	3.7	United Kingdom	39677	3.2	Philippines	14292	3.8	Turkey	390127	3.8	South Korea	1000637	5.2
Sri Lanka	16363	2.3	Iran	31312	2.5	Japan	12599	3.4	Saudi Arabia	266320	2.6	China	866419	4.5
Malaysia	15838	2.2	South Korea	30973	2.5	United States	12360	3.3	Philippines	239269	2.4	Germany	736608	3.8
Vietnam	14195	2.0	France	29700	2.4	Germany	12266	3.3	Thailand	208180	2.1	France	560047	2.9
Iraq	13587	1.9	Colombia	25912	2.1	South Africa	11600	3.1	Kuwait	202960	2.0	Brazil	401382	2.1
South Korea	11816	1.7	Sri Lanka	21892	1.8	South Korea	9788	2.6	South Africa	196602	1.9	Philippines	339139	1.8
Sudan	11147	1.6	United Arab Emirates	20856	1.7	Samoa	8402	2.3	United States	170199	1.7	Taiwan	321358	1.7
Thailand	10306	1.5	Algeria	18829	1.5	Malaysia	6506	1.7	Iran	166623	1.6	Australia	284135.5	1.5
Singapore	9792	1.4	Morocco	18717	1.5	Ireland	5526	1.5	Ukraine	163909	1.6	Russia	278332	1.4
Afghanistan	9774	1.4	Romania	17875	1.4	Canada	5425	1.5	Bangladesh	162765	1.6	Colombia	268298	1.4
Indonesia	8720	1.2	Germany	16358	1.3	France	4436	1.2	Sri Lanka	159197	1.6	Italy	258892	1.3
Fiji	8515	1.2	Bangladesh	15083	1.2	Taiwan	3875	1.0	Australia	154676	1.5	Spain	227517	1.2
Myanmar	7860	1.1	Mexico	14843	1.2	Tonga	3770	1.0	Taiwan	152157	1.5	Venezuela	213125	1.1
United States	7710	1.1	Lebanon	14788	1.2	Thailand	3722	1.0	Egypt	143768	1.4	Israel	212934	1.1
Pakistan	7301	1.0	Russia	14656	1.2	Sri Lanka	2441	0.7	Ghana	121162	1.2	Turkey	212565	1.1

Total	710627		Total	1250055		Total	373299		Total	10173350		Total	19277671	

^*∗*^Reports list New Zealand as the top source for New Zealand.

**Table 2:** Top source countries for foreign-born TB cases, 2005–2009.

Australia			Canada			New Zealand			United Kingdom			United States		
Country	Number	%	Country	Number	%	Country	Number	%	Country	Number	%	Country	Number	%
India	1004	19.9	India	912	17.1	India	285	26.0	India	7009	24.4	Mexico	9098	24.1
Vietnam	551	10.9	China	795	14.9	China	124	11.3	Pakistan	4811	16.7	Philippines	4302	11.4
China	433	8.6	Philippines	759	14.3	Philippines	75	6.8	Somalia	3056	10.6	Vietnam	2885	7.7
Philippines	406	8.1	Vietnam	352	6.6	Samoa	58	5.3	Bangladesh	1229	4.3	India	2869	7.6
Indonesia	246	4.9	Pakistan	190	3.6	South Korea	55	5.0	Zimbabwe	1222	4.3	China	2019	5.4
Papua New Guinea	170	3.4	Ethiopia	149	2.8	South Africa	35	3.2	Nigeria	905	3.2	Guatemala	1154	3.1
Sudan	159	3.2	Somalia	144	2.7	Cambodia	35	3.2	Kenya	653	2.3	Haiti	1067	2.8
Nepal	123	2.4	Haiti	131	2.5	New Zealand^*∗*^	33	3.0	South Africa	560	2.0	Ethiopia	887	2.4
United Kingdom	97	1.9	Sri Lanka	110	2.1	Somalia	30	2.7	Philippines	543	1.9	Honduras	853	2.3
Somalia	96	1.9	Unknown	106	2.0	Tonga	30	2.7	Afghanistan	457	1.6	South Korea	832	2.2
Sri Lanka	88	1.8	South Korea	103	1.9	Malaysia	25	2.3	Uganda	446	1.6	Somalia	786	2.1
Bangladesh	88	1.8	Afghanistan	85	1.6	United Kingdom	21	1.9	Sri Lanka	440	1.5	El Salvador	713	1.9
Myanmar	85	1.7	Bangladesh	79	1.5	Indonesia	21	1.9	China	421	1.5	Peru	692	1.8
Cambodia	84	1.7	Republic of Congo	73	1.4	Vietnam	20	1.8	Nepal	414	1.4	Ecuador	601	1.6
Pakistan	79	1.6	Sudan	60	1.1	Fiji	20	1.8	Eritrea	390	1.4	Cambodia	475	1.3
Afghanistan	78	1.6	Italy	53	1.0	Thailand	19	1.7	Ethiopia	274	0.95	Dominican Republic	434	1.2
Thailand	75	1.5	Cambodia	52	1.0	Myanmar	18	1.6	Republic of Congo	272	0.95	Pakistan	407	1.1
Ethiopia	74	1.5	Nepal	51	1.0	Ethiopia	18	1.6	Jamaica	246	0.86	Kenya	366	0.97
Malaysia	57	1.1	Kenya	45	0.8	Zimbabwe	15	1.4	Ghana	246	0.86	Myanmar	349	0.93
New Zealand	54	1.1	Eritrea	44	0.8	Kiribati	13	1.2	Zambia	223	0.78	Laos	344	0.91

Total	5036		Total	5327		Total	1098		Total	28760		Total	37684	

^*∗*^New Zealand categorizes TB cases among non-New Zealand citizens as foreign-born. Thus, any persons born in New Zealand who are not New Zealand citizens but are diagnosed with TB will be included in the surveillance system among the foreign-born cases.

**Table 3:** Top source countries for foreign-born MDR TB cases, 2005–2009.

Australia			Canada			New Zealand			United Kingdom			United States		
Country	Number	%	Country	Number	%	Country	Number	%	Country	Number	%	Country	Number	%
Papua New Guinea	42	42.4	China	12	16.4	China	6	40.0	India	61	27.9	Mexico	66	13.7
India	19	19.2	India	10	13.7	South Korea	2	13.3	Somalia	27	12.3	Philippines	56	11.6
Vietnam	8	8.1	Philippines	8	11.0	New Zealand	1	6.7	Pakistan	19	8.7	India	51	10.6
China	7	7.1	Vietnam	7	9.6	Cambodia	1	6.7	Lithuania	10	4.6	Vietnam	47	9.8
Indonesia	3	3.0	Peru	6	8.2	South Africa	1	6.7	Nigeria	9	4.1	China	32	6.6
Philippines	3	3.0	Afghanistan	3	4.1	Indonesia	1	6.7	Nepal	8	3.7	Peru	24	5.0
United Kingdom	3	3.0	Russia	3	4.1	Peru	1	6.7	China	7	3.2	Laos	21	4.4
Thailand	2	2.0	Nigeria	3	4.1	Philippines	1	6.7	Zimbabwe	7	3.2	South Korea	14	2.9
Uzbekistan	2	2.0	South Korea	3	4.1	India	1	6.7	Bangladesh	6	2.7	Guatemala	14	2.9
Myanmar	1	1.0	Ukraine	2	2.7				Afghanistan	4	1.8	Ethiopia	13	2.7
Sudan	1	1.0	Ethiopia	2	2.7				South Africa	4	1.8	Dominican Republic	12	2.5
Ethiopia	1	1.0	Nepal	2	2.7				Philippines	4	1.8	Russia	11	2.3
Nigeria	1	1.0	Somalia	2	2.7				Kenya	3	1.4	Nepal	11	2.3
Cambodia	1	1.0	Guyana	2	2.7				Kazakhstan	3	1.4	Myanmar	10	2.1
Pakistan	1	1.0	Sudan	1	1.4				Cote d'Ivoire	3	1.4	Thailand	10	2.1
Somalia	1	1.0	Cambodia	1	1.4				Zambia	3	1.4	Haiti	10	2.1
East Timor	1	1.0	South Africa	1	1.4				Angola	3	1.4	Somalia	8	1.7
Sierra Leone	1	1.0	Pakistan	1	1.4				Latvia	3	1.4	Sudan	7	1.5
Lebanon	1	1.0	Kenya	1	1.4				Ukraine	2	0.91	Ecuador	7	1.5
Ukraine	0	0.0	Malaysia	1	1.4				Thailand	2	0.91	Moldova	4	0.83

Total	99		Total	73		Total	15		Total	219		Total	482	
